# COVID-19 and Regional Income Inequality in China

**DOI:** 10.3389/fpubh.2021.687152

**Published:** 2021-05-11

**Authors:** Jianfu Shen, Wai Yan Shum, Tsun Se Cheong, Lafang Wang

**Affiliations:** ^1^Department of Building and Real Estate, Hong Kong Polytechnic University, Kowloon, Hong Kong; ^2^Department of Economics and Finance, Hang Seng University of Hong Kong, Hong Kong, China; ^3^School of Economics and Trade, Hunan University, Changsha, China

**Keywords:** regional income inequality, distribution dynamics, China, COVID, rural-urban disparity

## Abstract

This study investigates the impact of COVID-19 and social distancing policies on regional income inequality. We base our study on a sample of 295 prefecture (and above) cities in 31 provinces in China. A distribution dynamics approach is employed to reveal the trend and movement of disposable income per capita in each city before the COVID-19 pandemic, during the COVID-19 pandemic, and in the period when the COVID-19 was under the control. The findings reveal significant negative economic consequences of the COVID-19 in the first quarter of 2020 and show that most cities will converge to a level of disposable income which is much less than the Pre-COVID level if the COVID pandemic persists. Regional income inequality has intensified in the cities that have a longer duration of stringent social distancing policies during the COVID-19 pandemic and disappeared in the cities with policies of short duration. Disposable income per capita for urban residents recovered quickly when the transmission of coronavirus was effectively contained; and yet the impact of the pandemic on rural residents remains unresolved, if not intensified. This study demonstrates a significant divergence of the trend of disposable income across cities with different durations of social distancing policies and between urban and rural residents. It also highlights the importance of stringent social distancing policies in containing the spread of virus in a short time and calls for special policy attention for rural regions in the recovery from the COVID-19.

## Introduction

Up to March 2021, the COVID-19 pandemic has infected millions and caused millions of deaths across the globe^1^. Many countries have adopted strict social distancing policies, including travel restriction, school closure and even the lockdown policies (1). The pandemic also lead to substantial economic loss: according to the data from International Monetary Fund (IMF), the GDP growth in 2020 is a negative 4.4% in the world, a negative 5.8% in the advanced economies, and a negative 3.3% in emerging market and developing economies^2^. Some studies show that the COVID-19 crisis may intensify the income inequality across the groups of employees by gender, education, earnings level and ethnicity and working style (2, 3), and cross countries (4). However, how COVID-19 crisis affects income inequality within countries has not been explored, although different regions in a country may expose to different levels of coronavirus transmission risks, adopt different policies to contain the spread of the virus, have different capacity to cope with the outbreak of the virus and suffer the different mortality and economic loss. This study fills this gap by investigating the impacts of COVID-19 and the associated social distancing policies on regional income inequality in China.

Whether COVID-19 intensifies or mitigates regional income inequality in China is an interesting question for several reasons. First, despite that overall income inequality could decline after four decades of rapid economic growth, urban and rural income equalities across regions, e.g., eastern region vs. western region, are widened due to the gaps in economic development, industrial structure, urban-rural divide, fiscal capacity, and others [e.g., Xie and Zhou (5)]. COVID-19 may cause more loss to low-income regions as they have weak fiscal capacity and lower government efficiency to deal with the crisis. On the other hand, high-income regions have closer connections with Wuhan, the epicenter of COVID-19, and are more likely to have more infections and deaths as well as aggressive social distancing policies than low-income regions (4). Thus, the impact of COVID-19 on income inequality between high-income and low-income regions remain an empirical question that we explore in this study.

We explore the effect of COVID-19 on city-level income inequality using a sample of 295 prefecture (and above) cities in 31 provinces^3^. Our sample contains disposable income per capita, disposable income per capita of urban households, and disposable income per capita of rural residents in these cities in 2019 and 2020. We focus on the disposable incomes before the outbreak of COVID-19 (2019Q4), the quarter of outbreak (2020Q1), and the quarters after the coronavirus was under control in most of regions (2020Q2 and 2020Q3)^4^. All 31 provinces had launched a Level I Emergency Response Situation (ERS) in the late January, 2020; from then stringent social distancing policies, including the policies of “closed management of communities” and “family outdoor restrictions,” had been implemented in more than 250 prefecture cities outside Hubei (6, 7). In late February 2020, some cities downgraded the ERS to Level II or even Level III after the pandemic had relieved, and while the top ERS remained in some cities. According to the duration of the Level I ERS, we divide the cities in our sample into three groups and explore the potential impacts of COVID-19 on income inequality in cities with different response policies.

We apply a distribution dynamics approach to investigate the trend and movement of disposable incomes across cities with different levels of social distancing policies and between rural and urban regions. The dynamics of quarterly disposable income per capita (all residents, urban households and rural residents) in relative to the average value of disposable income in all the cities are presented in the pre-COVID period, during the COVID period and in the post-COVID period. The findings show that disposable incomes per capita in all cities significantly reduce during the COVID period and the income inequality across cities was intensified in the cities with longer duration of Level I ERS. Most cities would converge to a level of disposable income which is much less than the pre-COVID level if the pandemic persists. We find that the disposable incomes of urban residents recover quickly from the second quarter of 2020; and yet the negative shock of the pandemic on the disposable income of rural residents remains unresolved in the post-COVID period. Rural residents are more severely affected by the COVID-19 pandemic and the social distancing policies (2, 8).

This study contributes to two streams of literature: regional income inequality in China and the economic consequences of the COVID-19. Previous studies show that the income inequality between coastal and inland regions, and between rural and urban regions worsens since mid-1980s [e.g., (5, 9)], although it is plateauing in recent years (10). Our results indicate that the pandemic and the duration of stringent social distancing policies affect the regional income inequality. We are also the first study that explores the effect of the COVID-19 pandemic and the social distancing policies on the income inequality within a country. Deaton (4) shows that global income inequality across countries decreased in 2020 because rich countries suffered more deaths and larger declines in income per capita. Our study shows that the regional income inequality could be intensified due to the economic consequence of the COVID-19, especially for rural residents.

## Background and Literature Review

### COVID-19 Pandemic in China

The outbreak of COVID-19 started in the late January 2020 in China, spreading from Wuhan, the capital of Hubei Province and the most populous city in central region of China to all 31 provinces. The numbers of confirmed cases arose fast during the period of the last week of January 2020 and the first 2 weeks of February 2020. The numbers of new daily cases in Hubei Province reached a peak of 14,840 on February 12 when the Chinese Center for Disease Control and Prevention (CDC) began to include clinically diagnosis. The peak of daily confirmed cases in the 30 non-Hubei provinces occurred on February 3, 2020 with a number 890. The epidemic curve had been significantly flattened after the third week of February, 2020; and most provinces outside Hubei had effective contained the transmission of coronavirus. The number of new daily cases outside Hubei fall to a one digit 9 on February 24, 2020, although there were several waves of infections in many cities after the first quarter of 2020^5^. Around 3 weeks later, the numbers of new daily cases in Hubei also fall to one digit or even zero in the mid-March 2020; and the situation of low or zero infections maintained subsequently. Appendix 1 presents the numbers of new daily cases in overall 31 provinces, in Hubei and in non-Hubei provinces from January 20, 2020 to December 30, 2020.

Flattening the epidemic curve in < 6 weeks in most regions in China could be attributed to a systematic and quick government response to the outbreak. Provinces that are geographically close to Wuhan launched Level I Emergency Response Situation on or before January 24, 2020, followed by announcements in next day from cities located in the western and northern regions of the country^6^. Systematic intervention measures from central government, including social distancing policies, quarantine strategies, tracing and managing close contracts of COVID-19 confirmed cases, etc., were executed by local governments. While Wuhan and other cities in Hubei were completely shut down after January 23, 2020, some cities outside Hubei were also partially shut down (6) and more than 250 prefecture level cities had implemented very stringent social distancing policies such as closed management of communities and family outdoor restrictions (7). Public transportation was suspended in cities; residents were restricted to enter and exit communities; and only one family member was allowed to go outside once every 1 or 2 days. The lockdown policies lasted for several days to several weeks in different cities. The human mobility gradually returned to normally when the travel restrictions were removed. Three provinces, i.e., Guangdong, Jiangsu and Shanxi, downgraded the ERS to Level II on February 24, 2020; and some remote provinces, such as Gansu, Guizhou, and Yunan issued a downgrade to Level II ERS from February 21, 2020. Table 1 reports the numbers of confirmed cases on January 23, 2020 and in the first quarter of 2020 in 31 provinces and the dates to announce Level I ERS and downgrade to Level II/II.

**Table 1:** The numbers of confirmed cases, ESR announcement dates, and durations of Level I ERS in 31 provinces.

**Province**	**No. of cases on Jan 23**	**No. of cases in 2020Q1**	**Level I ERS Date**	**Downgrade to Level II**	**Downgrade to Lever III**	**Days of Level I**	**Level I Duration**
Hubei	549	66,907	24/1/2020	2/5/2020	13/6/2020	99	Long
Beijing	26	413	24/1/2020	30/4/2020	6/6/2020	97	Long
Hebei	2	318	24/1/2020	30/4/2020	6/6/2020	97	Long
Tianjin	7	136	24/1/2020	30/4/2020	6/6/2020	97	Long
Shanghai	20	337	24/1/2020	24/3/2020	9/5/2020	60	Long
Henan	9	1,272	25/1/2020	19/3/2020	5/5/2020	54	Long
Jiangxi	7	935	24/1/2020	12/3/2020	20/3/2020	48	Long
Shandong	9	756	24/1/2020	12/3/2020	20/3/2020	48	Long
Hunan	24	1,018	23/1/2020	10/3/2020	31/3/2020	47	Long
Chongqing	27	576	24/1/2020	10/3/2020	24/3/2020	46	Long
Heilongjiang	2	480	25/1/2020	4/3/2020	25/3/2020	39	Medium
Tibet	0	1	27/1/2020	6/3/2020		39	Medium
Zhejiang	43	1,205	23/1/2020	2/3/2020	23/3/2020	39	Medium
Ningxia	2	73	25/1/2020	28/2/2020	6/5/2020	34	Medium
Qinghai	0	18	26/1/2020	–	26/2/2020	34	Medium
Shaanxi	3	245	25/1/2020	–	28/2/2020	34	Medium
Fujian	5	296	24/1/2020	26/2/2020	26/2/2020	33	Medium
Sichuan	15	538	24/1/2020	26/2/2020	25/3/2020	33	Medium
Anhui	15	990	24/1/2020	25/2/2020	15/3/2020	32	Short
Guangdong	53	1,349	23/1/2020	24/2/2020	9/5/2020	32	Short
Hainan	8	168	25/1/2020	–	26/2/2020	32	Short
Jilin	3	93	25/1/2020	26/2/2020	20/3/2020	32	Short
Guangxi	13	252	24/1/2020	–	24/2/2020	31	Short
Inner Mongolia	1	75	25/1/2020	–	25/2/2020	31	Short
Xinjiang	2	76	25/1/2020	25/2/2020	7/3/2020	31	Short
Yunnan	2	174	24/1/2020	–	24/2/2020	31	Short
Guizhou	3	146	24/1/2020	–	23/2/2020	30	Short
Jiangsu	9	631	25/1/2020	24/2/2020	27/3/2020	30	Short
Shanxi	0	133	25/1/2020	24/2/2020	10/3/2020	30	Short
Liaoning	4	122	25/1/2020	–	22/2/2020	28	Short
Gansu	2	91	25/1/2020	–	21/2/2020	27	Short

*ESR announcement dates are collected from official websites. The numbers of confirmed cases are from China CDC*.

Hubei was the last province that issued a downgrade of ERS to Level II on May 2, 2020. The duration of Level I ERS is as long as 99 days, followed by 97 days in Beijing-Tianjin-Hebei integration area. According to the numbers of days with Level I ERS, we divide the cities in our sample into three groups: long duration group, medium duration group and short duration group. Studies in COVID-19 show that strong social distancing policies such as lockdowns increase unemployment (11), reduce consumer spending (12, 13) and temporarily improve air quality due to the restriction of human mobility (14, 15). Cities with longer duration of Level I ERS maintained stringent social distancing policies for a longer time, and hence could suffer more negative economic impacts from COVID-19. On the other hand, cities in the cities with short duration of Level I ERS could recover from recession faster. The changes of economic outputs and disposable incomes in Chinese cities may be affected by the duration of Level I ERS.

Table 2 shows the statistics of cities in the three groups by the durations of Level I ERS. The numbers of cities in the groups of short duration, medium duration and long duration are 141, 71, and 84, respectively. Cities in the short duration group have lower GDP growth rate and GDP per capita in 2019 than cities in medium and long durations, indicating that these cities are located in relatively low-income regions; and while cities with longest duration have largest GDP growth rate and GDP per capita. The long duration group contains more cities from eastern and central parts of China, as these cities had closer connections with Wuhan and on average suffered to more infections. Cities located in western region are more likely to have short duration or medium duration of Level I ERS.

**Table 2:** The number of cities, average GDP growth in 2019, average GDP per capita in 2019, and located regions in three groups by duration of Level I ERS.

**Group**	**No. of cities**	**2019 GDP growth**	**2019 GDP per capita**	**Eastern (%)**	**Central (%)**	**Western (%)**	**Northeastern (%)**
Short duration	141	6.80%	61,804	26.24%	19.15%	39.01%	15.60%
Medium duration	71	7.41%	64,845	28.17%	0.00%	54.93%	16.90%
Long duration	84	8.07%	65,066	35.71%	63.10%	1.19%	0.00%

*Eastern region includes Beijing, Tianjin, Shanghai, Fujian, Guangdong, Hainan, Hebei, Jiangsu, Shandong, Zhejiang; central region includes Anhui, Jiangxi, Henan, Hubei, Hunan, and Shanxi; western region includes Chongqing, Gansu, Guangxi, Guizhou, Inner Mongolia, Ningxia, Qinghai, Shaanxi, Sichuan, Tibet, Xinjiang, and Yunnan; and northeast western includes Liaoning, Jilin, and Heilongjiang. GDP data are from CEIC*.

### Literature Review

Regional income inequality, including the disparities between coastal and inland regions, and urban and rural regions, in China has been widely studied in the literature. Several studies document that after the reform from 1978, regional income inequality first declined and then widened as coastal cities in the eastern regions grew much faster than cities in the central and western regions (16, 17). The regional income disparity can be attributed to geographical advantages/disadvantages (18), fiscal decentralization (9), labor mobility and urbanization (18, 19), decreasing labor share and rising profit share (20), migration and city population size (21), etc. Xie and Zhou (5) show that income inequality in China since 2005 can be largely explained by regional disparities across cities and rural-urban divide. The inequality between urban and rural areas is caused by urban-biased economic policies in China (22, 23). A recent study by Kanbur et al. (10) shows that inequalities between rural and urban areas or coastal and inland regions started to plateau or even decline after 2008. They argue that the decreasing inequality could be explained by the lower rural-urban wage differentials, infrastructure investment in rural regions, inequality-mitigating transfer and other government policies that benefit rural residents.

The COVID-19 pandemic has caused a large amounts of deaths and brought substantial economic loss to all countries. The economic consequences of COVID-19 and associated social distancing policies may vary across groups and intensify income inequality. Bonaccorsi et al. (24) find that low-income individuals in Italy are exposed more to the economic consequences of lockdown policies. The evidence from UK indicates low-income individuals are more likely to experience economic hardship than others during lockdown period (8). Young workers, self-employed workers and workers on low incomes are more likely to lose jobs or reduce earnings more than high-income workers during the lockdown (2); and working from home could intensify the existing inequalities in the labor market as it favors high-education and high-income individuals (2, 3). Overall, COVID-19 and the lockdown exacerbate income inequality between the poor and the rich.

Whether income inequality across regions in a country worsens has not been thoroughly examined. Deaton (4) shows that rich countries suffered more deaths in the COVID-19 pandemic and hence income per capita fell more than poor countries, despite that they had better health systems, higher incomes and better preparedness for the pandemic. The international income inequality decreased in 2020. It is not clear whether the regional income inequality, across cities and between urban and rural areas in China, widened due to the COVID-19. On the one hand, there were more confirmed cases and deaths in the regions with better economic development, which could cause longer duration of stringent social distancing policy (e.g., Level I ERS) and more potential economic loss. In this sense, income per capita could fall more in the rich regions (i.e., coastal regions and urban regions) than in the poor regions (i.e., inland regions and rural regions); and the regional income disparity may decline (4). On the other hand, poor regions may have lower fiscal capacity (24), poorer government efficiency (25), and less preparedness (4) to cope with the COVID-19 pandemic. Lower-income individuals in the poor regions may lose jobs or have more reductions of earnings because they were restricted to migrate to the rich regions. Apart from the question whether regional income inequality is affected by COVID-19, it is also important to know whether this potential effect is temporary which will disappear when the coronavirus is under the control, or it is a long-lasting effect. We examine these questions by analyzing the disposable income per capita of prefecture cities in China.

## Data and Methodology

### Data and Sample

Our sample contains 295 prefecture (and above) cities in China^7^. We collect quarterly city-level income data from CEIC database, including overall disposable income per capita, disposable income per capita for urban households, and disposable income per capita for rural residents^8^. Table 3 reports average disposable income per capita in each quarter of 2019 and 2020.

**Table 3:** Disposable income per capita by quarter in 2019 and 2020.

**Unit: RMB**	**2019Q1**	**2019Q2**	**2019Q3**	**2019Q4**	**2020Q1**	**2020Q2**	**2020Q3**	**2020Q4**
Disposable income	9023.23	7297.92	8035.04	7853.57	8761.19	7412.35	8310.62	8157.07
Disposable income: urban	10566.15	9068.06	10023.37	9809.43	10334.16	9227.04	10204.75	10308.53
Disposable income: rural	5429.44	3915.97	4702.84	4626.76	5039.11	3921.92	4810.21	5083.89

*Data source: CEIC*.

Table 3 shows that per capita disposable incomes for all residents, urban residents and rural residents all decreased in 2020Q1, in comparison with incomes in 2019Q1^9^. The quarter-to-quarter changes are −2.90, −2.20, and −7.19%, respectively. The results indicate that the COVID-19 pandemic negatively affect disposable incomes to all the residents, and the effect is much more pronounced for rural residents. The findings are consistent with previous studies (8, 24) that low-income individuals were more severely hit by the COVID-19 and the social distancing policies. As the spreading of coronavirus was effectively contained in China after the first quarter of 2020, disposable incomes for all residents, urban and rural residents slightly increase in the second quarter in comparison to 2019Q2. The disposable incomes continue to rise in the second half year of 2020 as the economy in China recovered from the recession. Despite the negative shock caused by the COVID-19 outbreak, the average per capita disposable incomes in 295 cities still increase in 2020^10^.

We are interested in examining the effect of the COVID-19 pandemic on regional income inequality. The analysis of the mobility of disposable income is separated into three-time episodes: the Pre-COVID period (from 2019Q3 to 2019Q4), the COVID period (from 2019Q4 to 2020Q1), and the Post-COVID period (from 2020Q1 to 2020Q2)^11^. To remove the seasonal pattern and the CPI effect on disposable incomes, we calculate a relative disposable income per capita (RDIPC) for all residents, urban residents and rural residents in a city. The relative disposable income per capita is calculated as the disposable income per capita in a city in quarter divided by the mean of 295 cities in that quarter. To investigate the varying impacts of social distancing policies, the cities in the sample are divided into three groups based on the duration of Level 1 ERS: long duration group, medium duration group, and short duration group.

### Methodology

In this study, the distribution dynamics approach is used to evaluate the impacts on the income distribution. Many scholars have studied the impacts of the pandemic by using econometrics techniques, however, it is notable that regression just cannot provide information on the evolution of the distribution across time. Therefore, a non-parametric method of stochastic kernels is employed in this study as we would like to understand the changes in the distribution in detail.

It is worth noting that distribution dynamics analysis is a very powerful tool as it can reveal the changes in distribution. Although the econometrics model can only provide a forecast of the dependent variable based on the changes in the independent variables, however, it is just impossible to predict the changes of distribution by using econometrics as the distribution is a two-dimensional entity while the econometrics model can only provide a point estimation of the data. As the major aim of this study is to examine the impacts on income distribution in China, so distribution dynamics analysis is a much better tool for this purpose. Moreover, this approach can also provide an estimation of the probability of the movement of the entities within the distribution, thereby unveiling the underlying trend of the changes of the distribution in detail.

The distribution dynamics analysis was first proposed by Quah (26) and it has been employed in many studies focusing on distribution, for example, Li and Cheong (27) and Zhang et al. (28). The stochastic kernel approach is used in this research because it can avoid the problem of distortion due to the discretization of data and it can also overcome the limitations of selection of the boundary values [please refer to Cheong and Wu (29) for details].

The kernel estimator can be represented by the following equation:

(1) $$\begin{array}{l} f_{t,t+\tau }\left(y,x\right)=\frac{1}{nh_{x}h_{y}}\sum_{i=1}^{n}K\left(\frac{x-x_{i}}{h_{x}},\frac{y-y_{i}}{h_{y}}\right) \end{array}$$

where *x*_*i*_ is the RDIPC value of a city at time *t* and *y*_*i*_ is the RDIPC value of that city at time *t* + *τ*. *h*_*x*_ and *h*_*y*_ are the bandwidth of variable of *x* and *y*, respectively. *K*(·) is the normal kernel function, *n* is the total number of transitions in the data. The optimal values of the bandwidth were determined by the procedures proposed by Silverman (30).

The conditional density function *g*_*τ*_ (*y*|*x*) can be computed by using *f*_*t*_ (*x*) and *f*_*t, t* + 1_ (*y, x*):

(2) $$\begin{array}{l} g_{\tau }\left(y|x\right)=\frac{f_{t,t+1}\left(y,x\right)}{f_{t}\left(x\right)} \end{array}$$

By using Equation (2) and repeating the process continuously, the ergodic distribution can be found. It is the steady state distribution in the long run. And it can be represented by the following:

(3) $$\begin{array}{l} f_{\infty }\left(y\right)=\int_{0}^{\infty }g_{\tau }\left(y|x\right)f_{\infty }\left(x\right)dx \end{array}$$

The ergodic distribution can provide vital information on the final distribution if the distribution dynamics remain unchanged. Given that the issue of COVID-19 was resolved very soon in China, so it is of interest to learn about the consequences and the impacts on China for a what-if scenario, namely, what will happen if the spread of the coronavirus went out of control. The ergodic distribution is very useful as it can be used to evaluate this scenario and reveal the impacts on income distribution in the long run when China cannot handle coronavirus. This finding is very important as it can disclose the details and the consequences for many developing countries with a large population.

Cheong and Wu (29) developed the mobility probability plot (MPP) and proposed a new framework in distribution dynamics analysis. Future movement of the cities within the distribution can be estimated by the MPP which is the probability of net upward movement of the cities. It is in the form:

(4) $$\begin{array}{l} p\left(x\right)=\int_{x}^{\infty }g_{\tau }\left(z|x\right)dz-\int_{0}^{x}g_{\tau }\left(z|x\right)dz \end{array}$$

The MPP is the probability of moving upward minus the probability of moving downward for the cities. A positive value of *p*(*x*) implies intuitively that the city will have a higher tendency to move up in the next period, and a negative value suggests that the city will have a higher tendency of moving downward in the next period. By observing the MPP, one can know the future movement of the cities at different levels of RDIPC. After the development of this model, it has been applied in different areas, including energy (31) and industrial output (29).

## Discussion

This section will present the distribution dynamics analysis of the economic impacts of the COVID-19 on regional income inequality in China. The findings can reveal the overall pattern of income distributions of all 295 cities before, during, and after the pandemic. The cities are divided into three groups based on the duration of Level 1 ERS: long, medium, and short duration. Urban and rural residents in a city of each duration groups are separately analyzed to see if the pandemic has different economic impacts on urban and rural residents.

### RDIPC of All Residents

#### RDIPC of All Residents in Cites With Long Duration of Level I ERS

**Figure 1:**
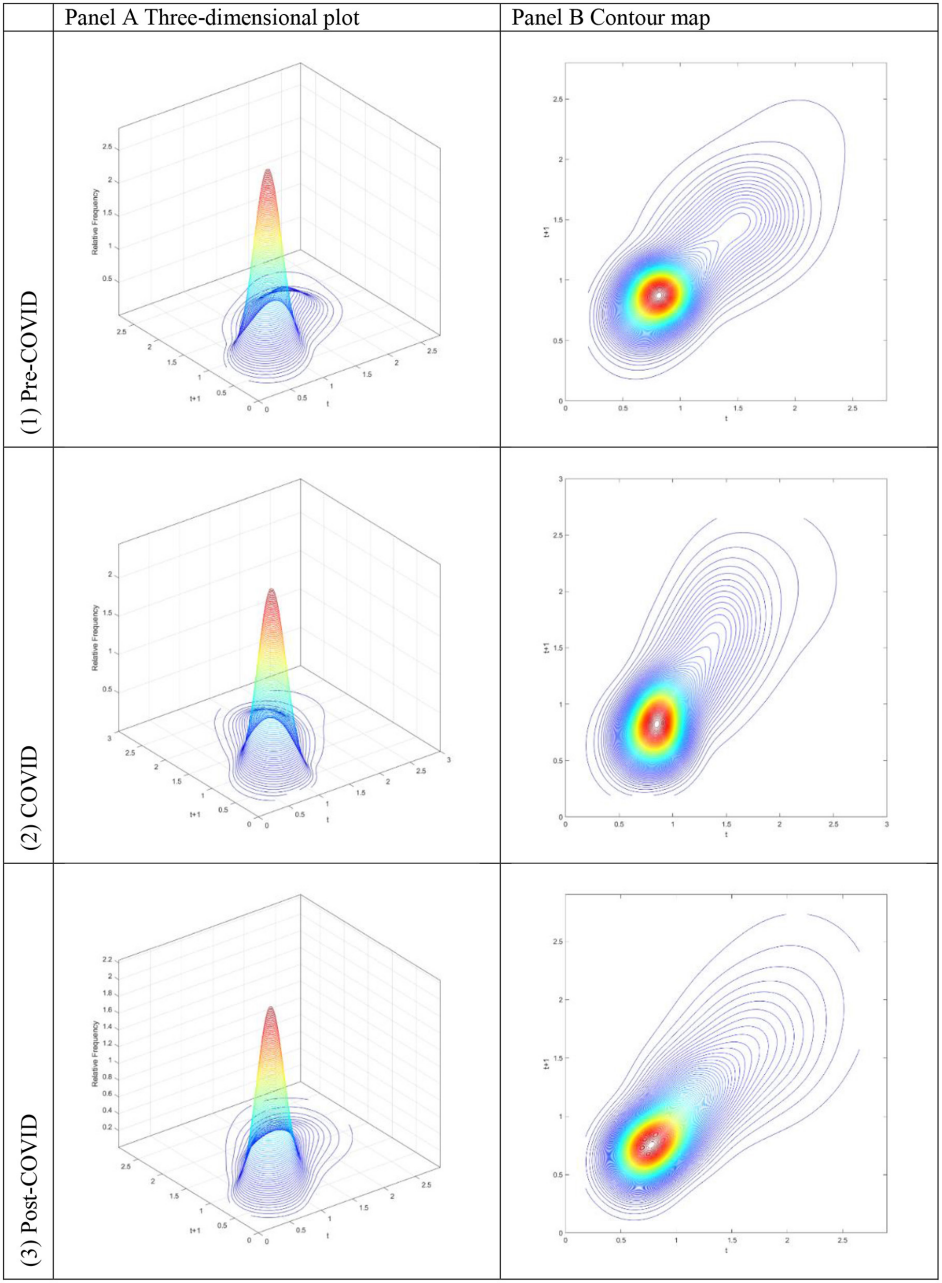
Three-dimensional plot **(A)** and Contour map **(B)** of transition probability kernel for the RDIPC of all residents in cities with the longest duration of Level I ERS with quarterly transitions during the (1) Pre-COVID, (2) COVID, and (3) Post-COVID periods. Source: authors' calculation.

The three-dimensional kernel-based transition probabilities for the RDIPC of all residents in cities with the longest duration of Level I ERS during the Pre-COVID, COVID, and Post-COVID periods are demonstrated in Figure 1A. The relative frequency—the height of a three-dimensional graph—shows the probability of transition at the province level from one specific RDIPC value in quarter *t* to another RDIPC value in quarter *t* + 1. Note that the RDIPC is measured relative to the global average; hence, the average of the RDIPC is one. It follows the measurement that a value less than one indicates a below-average RDIPC, whereas a value larger than one implies that the value is above average.

Along with the transition dynamics, contour maps for the RDIPC of all residents in cities with the longest duration of Level I ERS alert for three different periods are presented in Figure 1B. The contour maps provide the top views of the three-dimensional graphs. Thus, each vertical intersection of a contour map at period *t* denotes a probability density function, showing the transition probabilities from a particular RDIPC value at period *t* to another value at period *t*+*1*. For cities situated on the diagonal line, the RDIPC levels will remain the same before and after the transitions.

Figure 1B(1) shows that the peaks of the probability mass lie along the diagonal line during the Pre-COVID period. It implies that the RDIPCs of all residents in cities with the longest duration of Level I ERS tend to remain in their present positions without moving upwards or downwards in the transition dynamics. Two different probability mass concentrations of the transition probability can be observed; the tallest peak appears at around the RDIPC values of (0.8, 0.8), whereas the secondary peak appears at around the values of (1.5, 1.5). This pattern of concentrations indicates that while most cities have a slightly below-average RDIPC, a small group of cities has a high relative income per capita before the pandemic. Based on the relative income of all residents in each city, it is concluded that there is a noticeable imbalance in the economic development of cities with the longest duration of Level I ERS during the Pre-COVID period. Hence, it is clear from Figures 1A(1), 1B(1) that the economic development is uneven and a slow process in cities with the longest duration of Level I ERS.

As shown in the contour map for cities with the longest duration of Level I ERS in Figure 1B(2), it is obvious that the peaks of the probability mass no longer lie along the diagonal line during the COVID period; it tilted upward. It indicates that cities with a relatively high RDIPC value will have a greater tendency to move upwards from their present positions during the COVID period. Figure 1A(2) shows two different probability mass concentrations of the transition probability; the tallest peak appears at around the RDIPC values of (0.75, 0.75) and the secondary peak appears at around the values of (1.75, 2.25). It can be observed that the tallest peak appears at lower RDIPC values while the secondary peak appears at higher RDPIC values, in comparison to the peaks in the Pre-COVID period. Furthermore, cities with high RDIPC values show a greater tendency to move upward compared with those with low RDIPC values. These observations indicate that the pandemic has a negative economic impact on the cities with the longest duration of Level I ERS and the disparity that appeared during the Pre-COVID period has been intensified in the COVID period.

During the Post-COVID period, the contour lines of the probability mass are more condensed at around the RDIPC value of 1.5, as shown in the contour map for cities with the longest duration of Level I ERS in Figure 1B(3). Moreover, a single peak is located at around the RDIPC values of (0.7, 0.7) with no other peaks. It seems the disparity that appeared during the Pre-COVID and the COVID periods disappeared during the Post-COVID period.

The transition dynamics shown in Figure 1 contain a great deal of information. However, it is extremely hard to determine the location of the greatest portion of the probability mass in the three-dimensional plot or the contour map. Consequently, it is difficult to access a city's mobility in the disposable income (i.e., the probability of moving upwards or downward in the distribution). The mobility probability plot (MPP) can offer a direct interpretation of the probability mass in this regard.

The MPP marks the probability of net upward mobility as a percentage against the values of RDIPC. The net upward mobility ranges from −100 to 100; a positive value denotes that a city has a positive net probability of moving upward, while a negative value indicates that a city has a negative probability of moving up. Note that the MPP will intersect the horizontal axis whenever it moves from above the horizontal axis to the region below the horizontal axis. Thus, cities on the left-hand side of the intersection point have a positive chance to move upwards while cities on the right-hand side have a net probability of moving downwards. Consequently, these cities will congregate around the intersection points where the MPP moves from above the horizontal axis to the region below the horizontal axis. The intersection points will be referred to as the INTERSECTs hereafter. The transition dynamics underlying the MPP will eventually translate into a city-level long-run steady-state RDIPC distribution. In general, each INTERSECT in the MPP will translate into a peak appearing in the ergodic distribution, as cities will congregate around these INTERSECTs if the transition dynamics remain unchanged.

**Figure 2:**
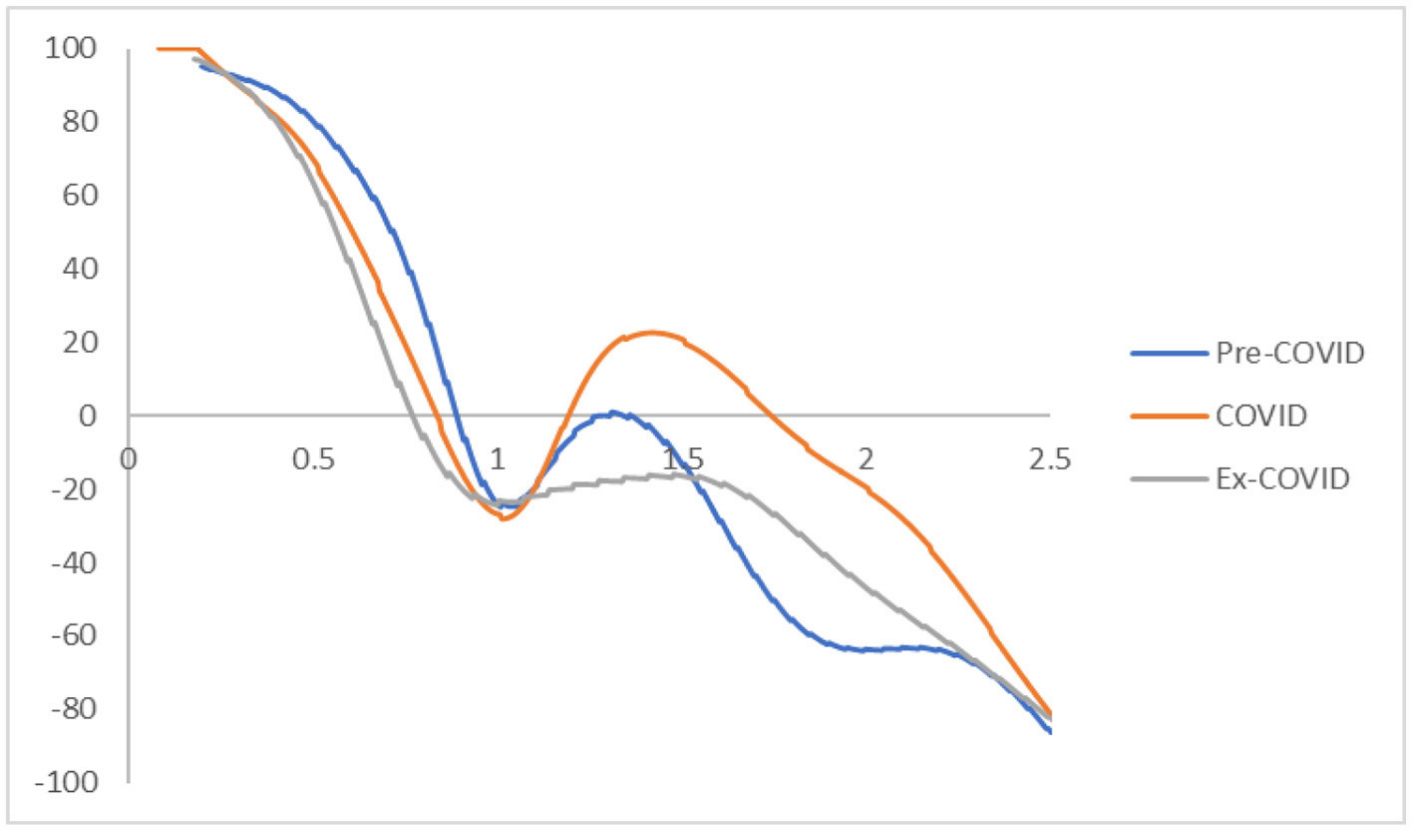
Mobility probability plot (MPP) for the RDIPC of all residents in cities with the longest duration of Level I ERS with quarterly transitions. Source: authors' calculation. N.B. The vertical axis indicates net upward mobility (%) and the horizontal axis indicates RDIPC values.

Figure 2 shows the MPPs of the RDIPCs of all residents in cities with the longest duration of Level I ERS during the Pre-COVID, COVID, and Post-COVID periods. The shape of the MPPs for three different periods in Figure 2 confirmed what can be observed from the transition dynamics in Figure 1. First, cities congregate roughly around the INTERSECTs. Second, the distance of the INTERSECTs is longer during the COVID period compared with the Pre-COVID period, indicating that the disparity has been intensified during the COVID period. Third, the disparity disappears during the Post-COVID period as the Post-COVID MPP has only one INTERSECT. As shown in Figures 1, 2, the transition dynamics underlying the MPP will eventually translate into a city-level long-run steady-state RDIPC distribution.

**Figure 3:**
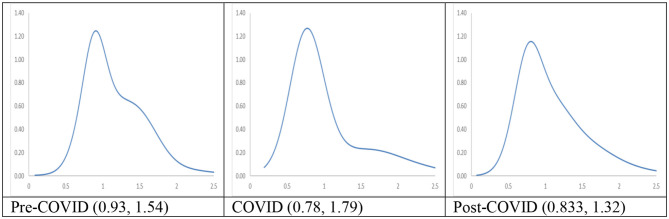
Ergodic distributions for the RDIPC of all cities with the longest Level I ERS duration. Source: authors' calculation. N.B. The vertical axis indicates the density of probability, the horizontal axis indicates RDIPC values, and the value of the peaks are in parentheses.

The long-run steady-state ergodic distributions for the cities with the longest duration of Level I ERS are shown in Figure 3. It can be observed that during the Pre-COVID period, many cities will converge toward an RDPIC value of 0.93—the highest peak that can be observed from the distribution when the transition dynamics in Figure 1 during the Pre-COVID period remain unchanged. Convergence club at a higher level can be found as a minor peak is located at the RDIPC value of 1.54. This implies that the disposable incomes of most cities will be remarkably close to the country mean, while the incomes for a few of them will be 1.5 times higher than the average. These values indicate that a certain degree of income disparity appears in these cities. These results are consistent with previous studies which showed that regional income inequality is severe in China [e.g., Xie and Zhou (5)]; and the disparity is large even in the cities located in provinces with relatively high GDP per capita, i.e., cities are with Level I ERS.

Figure 3 shows that as compared with the Pre-COVID ergodic distribution, the tallest peak of the COVID ergodic distribution shifted down from the RDPIC of 0.93–0.78, thus indicating that the COVID-19 and the social distancing policy have a significantly depressing effect on the disposable incomes in most cities. The secondary peak, on the other hand, shifted up from the RDPIC of 1.51–1.79. The downward movement of the tallest peak (from 0.93 to 0.78) indicates that the cities where RDIPC converges toward the country mean under the Pre-COVID dynamics will have the RDIPC converge below the country mean under the COVID dynamics. Furthermore, the upward movement of the secondary peak (from 1.51 to 1.79) along with the downward movement of the primary peak indicates that the pandemic intensifies income disparity among the cities with the longest duration of Level I ERS.

Figure 3 also shows the comparison of the Post-COVID ergodic distribution with the Pre-COVID and the post-COVID ergodic distributions. It can be observed that the tallest peak of the Post-COVID ergodic distribution is higher than that of the COVID ergodic distributions but less than that of the Pre-COVID ergodic distributions. Thus, it can be concluded that the adverse economic impacts brought by the pandemic diminished during the Post-COVID period, indicating a quick recovery from the COVID-19 recession in China. Secondly, a minor peak located near the RDIPC value of 1.32 can be vaguely observed. Since the distance between the convergence clubs during the COVID period (i.e., 0.78, 1.79) is larger than that of the Post-COVID period (i.e., 0.833, 1.32), the disparity in economic development triggered by the pandemic faded away.

In sum, we observe different mobility patterns of disposable incomes in cities with the longest duration of Level I ERS during the three periods. If the COVID dynamics persist, most cities will converge to RDIPC value which is less than the Pre-COVID level and disparity will be intensified due to the pandemic. However, the evidence shows that the cities with the longest duration of Level I ERS have recovered from the pandemic during the Post-COVID period, i.e., the convergence clubs have gradually restored to the Pre-COVID levels during the Post-COVID periods. It indicates that the prevention measures in these cities are effective.

#### RDIPC of All Residents in Cities With Medium Duration of Level I ERS

**Figure 4:**
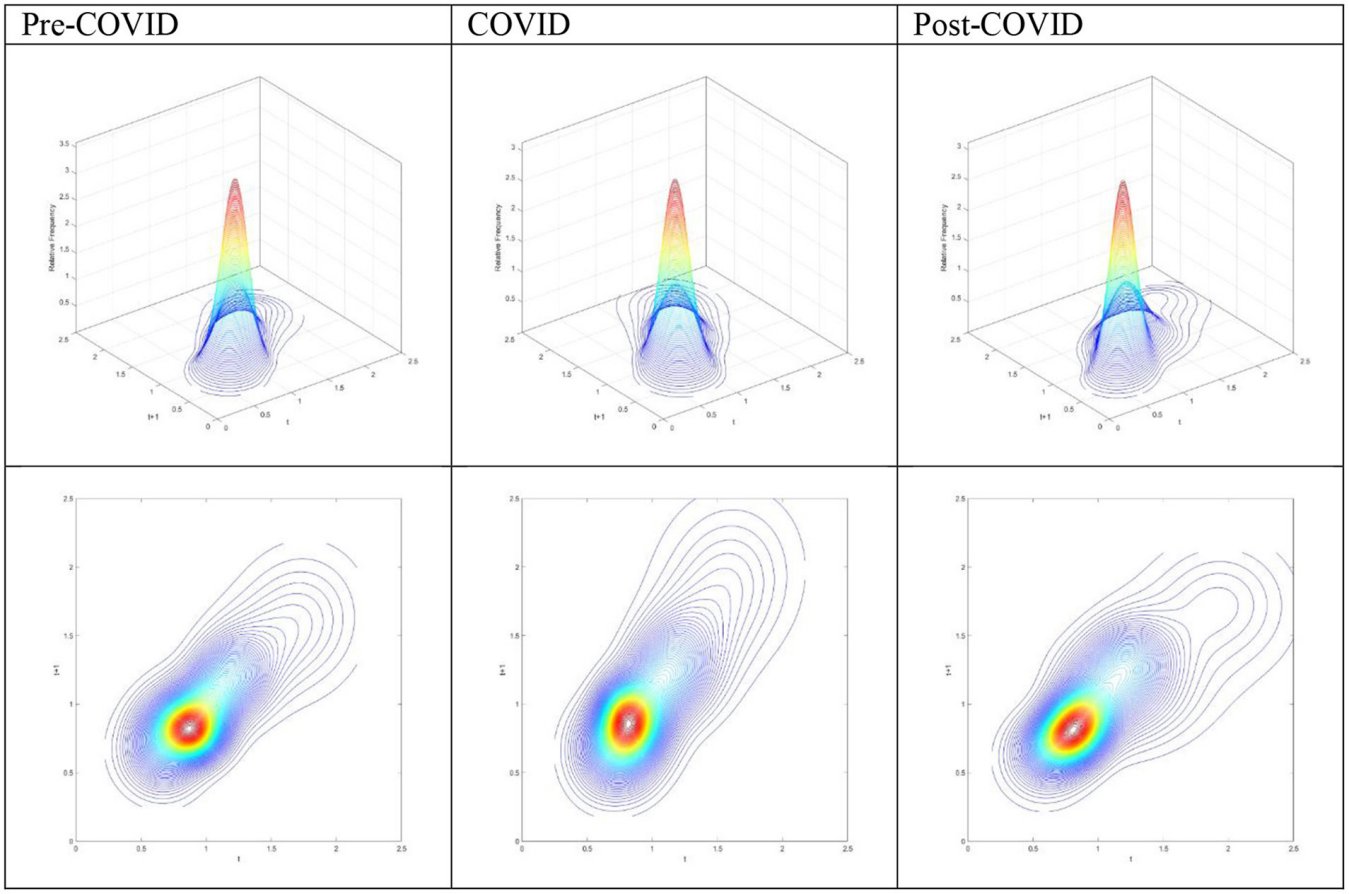
Three-dimensional plot and Contour map of transition probability kernel for the RDIPC of all residents in cities with medium duration of Level I ERS with quarterly transitions during the Pre-COVID, COVID, and Post-COVID periods. Source: authors' calculation.

The three-dimensional plots and the contour maps of RDIPC of all residents in cities with a medium duration of Level I ERS during the Pre-COVID, COVID, and Post-COVID periods are shown in Figure 4. As in the cities with the longest duration of Level I ERS, the peaks of the probability mass lie along the diagonal line during the Pre-COVID period. It indicates that the RDIPCs of all residents in cities with a medium duration of Level I ERS tend to remain in their present positions without moving upwards or downwards in the transition dynamics. During the COVID period, it is obvious that the peaks of the probability mass no longer lie along the diagonal line; it tilted upward. It indicates that during the COVID period, cities with a relatively high RDIPC value will have a greater tendency to move upwards from their present positions. The transition dynamics of the Post-COVID period share a similar shape with that of the Pre-COVID period.

**Figure 5:**
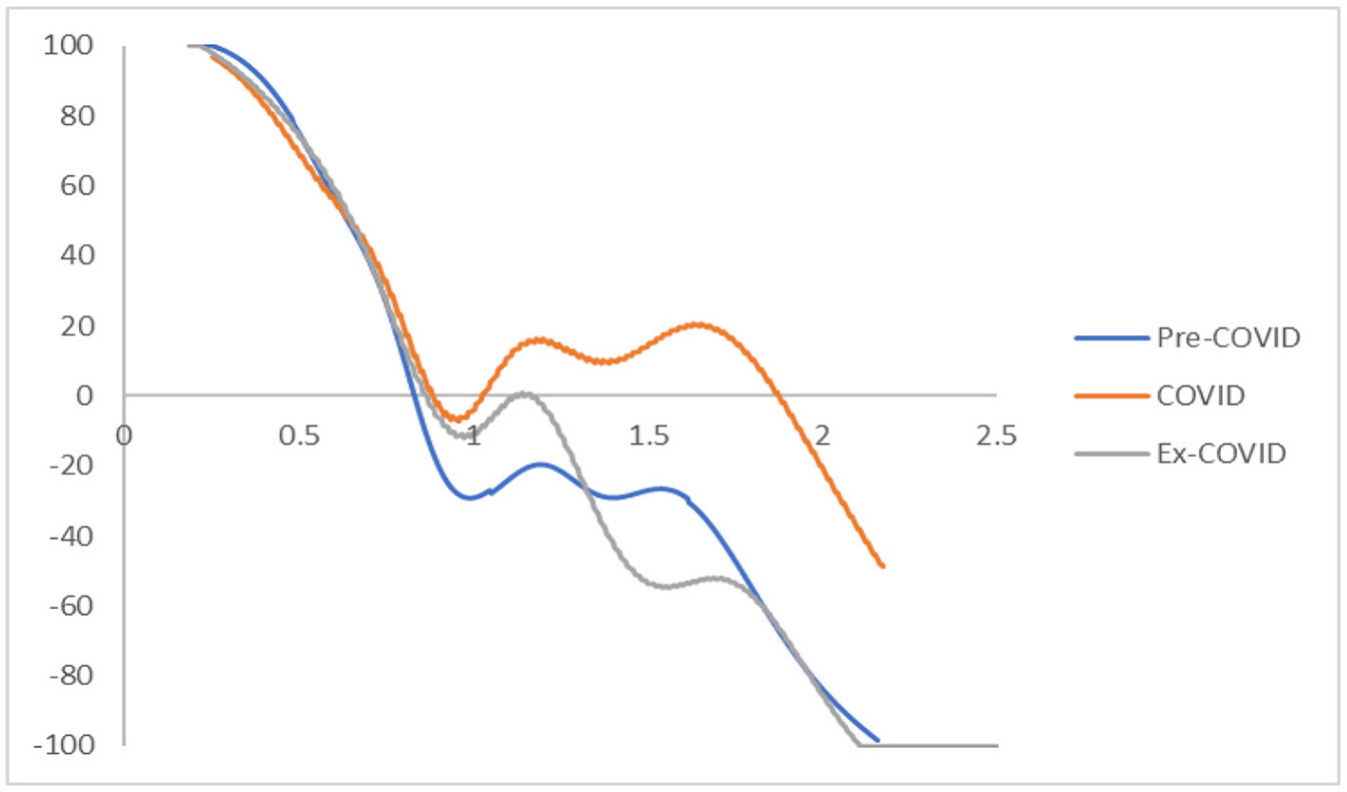
Mobility probability plot (MPP) for the RDIPC of all residents in cities with medium duration of Level I ERS with quarterly transitions. Source: authors' calculation. N.B. The vertical axis indicates net upward mobility (%) and the horizontal axis indicates RDIPC values.

The MPP of RDIPC of all residents in cities with a medium duration of Level I ERS during the Pre-COVID, COVID, and Post-COVID periods are shown in Figure 5. The shape of the MPPs confirmed what can be seen in the transition dynamics. During the COVID period, the MPP is above the horizontal line when the RDIPC value is above 1 and < 1.89, indicating that the cities with higher-than-average RDIPC values will have a positive chance to move upwards. The Pre-COVID and Ex COVID MPPs share a similar shape. It indicates that the transition dynamics of the Post-COVID period have been recovered to the Pre-COVID scenario.

**Figure 6:**
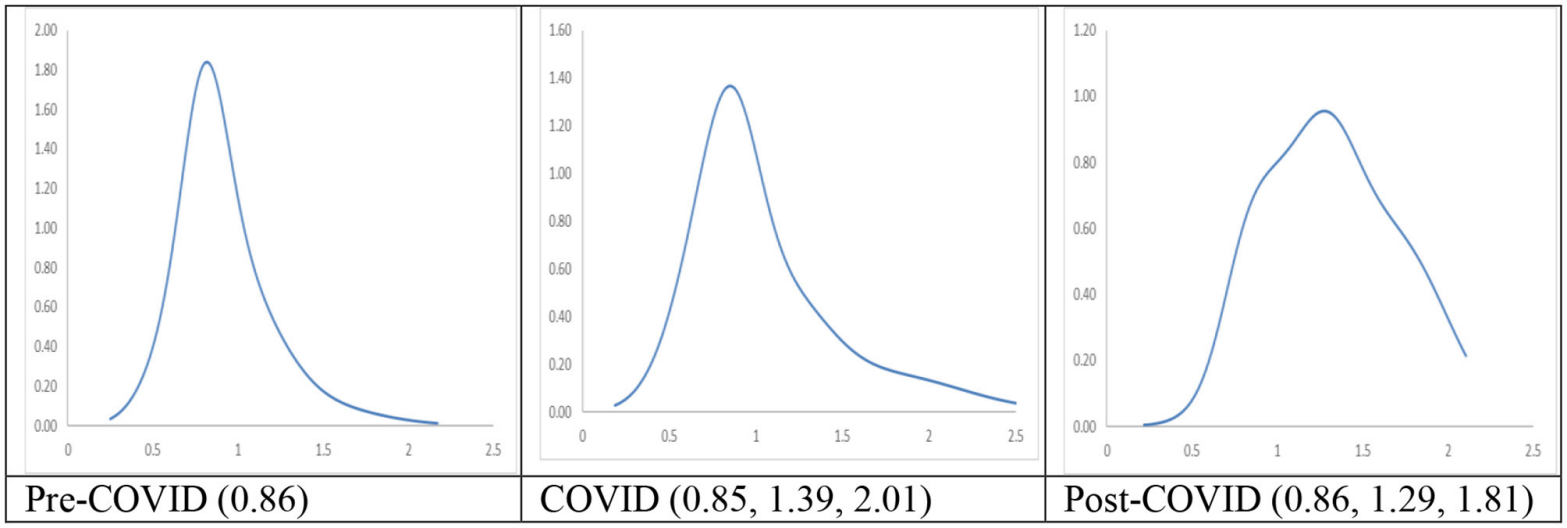
Ergodic distributions for the RDIPC of all cities with medium duration of Level I ERS. Source: authors' calculation. N.B. The vertical axis indicates the density of probability, the horizontal axis indicates RDIPC values, and the value of the peaks are in parentheses.

The long-run steady-state ergodic distributions and the MPP of RDIPC of all residents in cities with a medium duration of Level I ERS during the Pre-COVID, COVID, and Post-COVID periods are shown in Figure 6. If the Pre-COVID condition remains unchanged, it can be observed that most cities will converge to the RDIPC value of 0.86 with no other peaks. If the COVID condition persists, most cities will converge to the RDIPC value of 0.85, a remarkably similar figure to the Pre-COVID condition. However, compared with the Pre-COVID distribution, the COVID distribution is more dispersed, indicating that a larger proportion of cities will converge to a lower RDIPC value. As in the cities with the longest duration of Level I ERS, the disparity will be intensified if the COVID condition persists, as two minor peaks appear at the RDIPC values of 1.39 and 2.1, which, however, is not obvious. During the Post-COVID period, convergence clubs are located at the RDIPC values of 0.86, 1.29, and 1.81. Similar to the cities with the longest duration of Level I ERS, the distance of the convergence clubs is shorter during the Post-COVID than the COVID period. Thus, it can be concluded that pandemic-driven disparity fades away in the cities with a medium duration of Level I ERS. Additionally, the cities with a medium duration of Level I ERS recovered from the pandemic during the Post-COVID period.

#### RDIPC of All Residents in Cities With Short Duration of Level I ERS

We have shown that the mobility patterns of disposable incomes in the cities with long and medium duration of Level I ERS are similar. However, the economic impact of the pandemic on cities with the shortest duration of Level I ERS is not the same as in the cities with a long and medium duration of Level I ERS.

**Figure 7:**
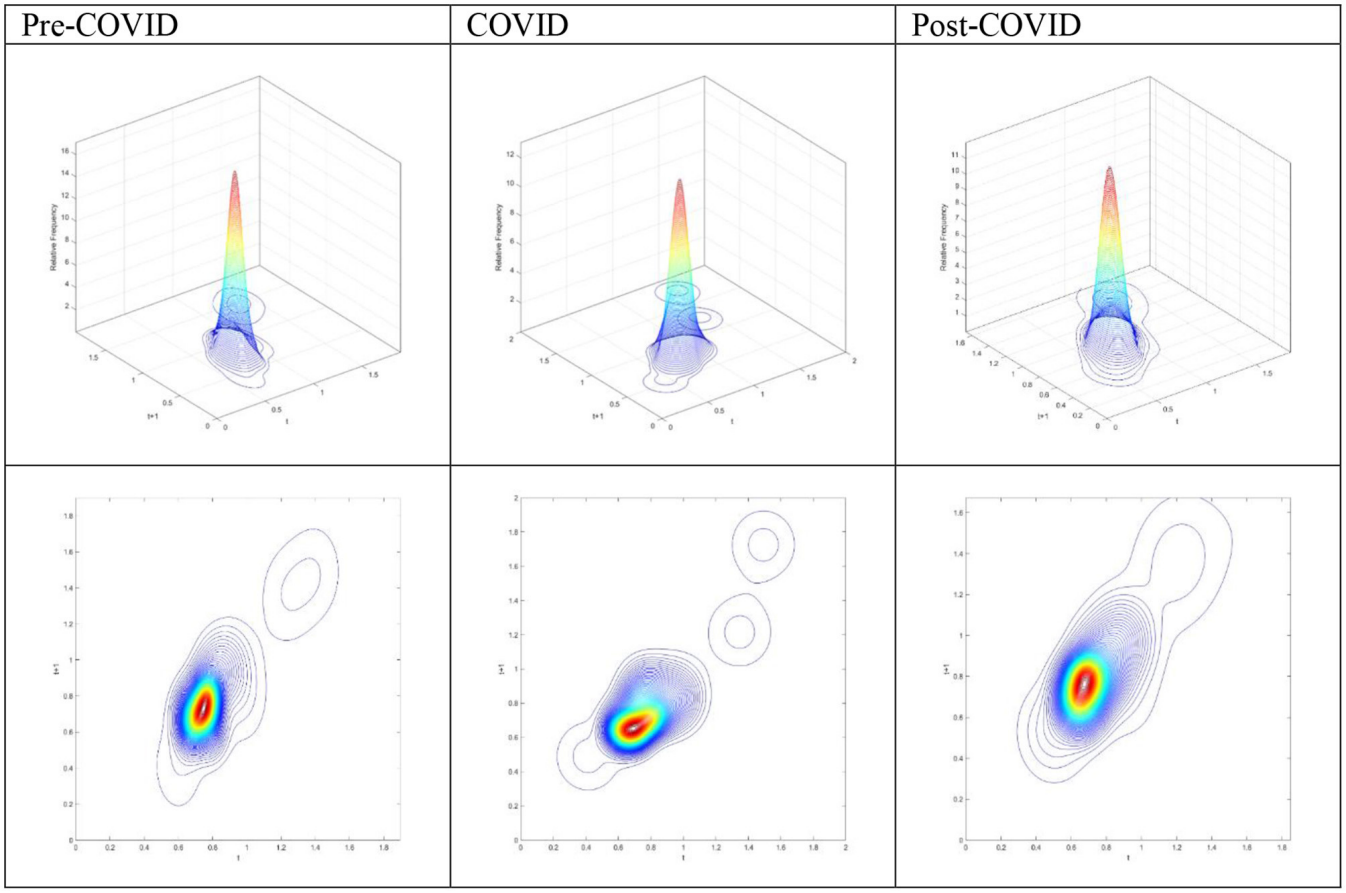
Three-dimensional plot and Contour map of transition probability kernel for the RDIPC of all residents in cities with the shortest duration of Level I ERS with quarterly transitions during the Pre-COVID, COVID, and Post-COVID periods. Source: authors' calculation.

The three-dimensional plots and the contour maps of RDIPC of all residents in cities with a short duration of Level I ERS during the Pre-COVID, COVID, and Post-COVID periods are shown in Figure 7. The three-dimensional plots and the contour maps for the Pre-COVID and Post-COVID periods share a similar shape. The peaks of the probability mass during the COVID period tilted downwards from the diagonal line as compared with the Pre-COVID condition when the RDIPC values are < 1.2. It indicates that the probability of a city with an RDIPC smaller than 1.2 to move upwards from its current position is less during the COVID period than the Pre-COVID and Post-COVID periods. However, when the RDIPC values are > 1.2, a small group of cities manages to move upwards from their present positions.

**Figure 8:**
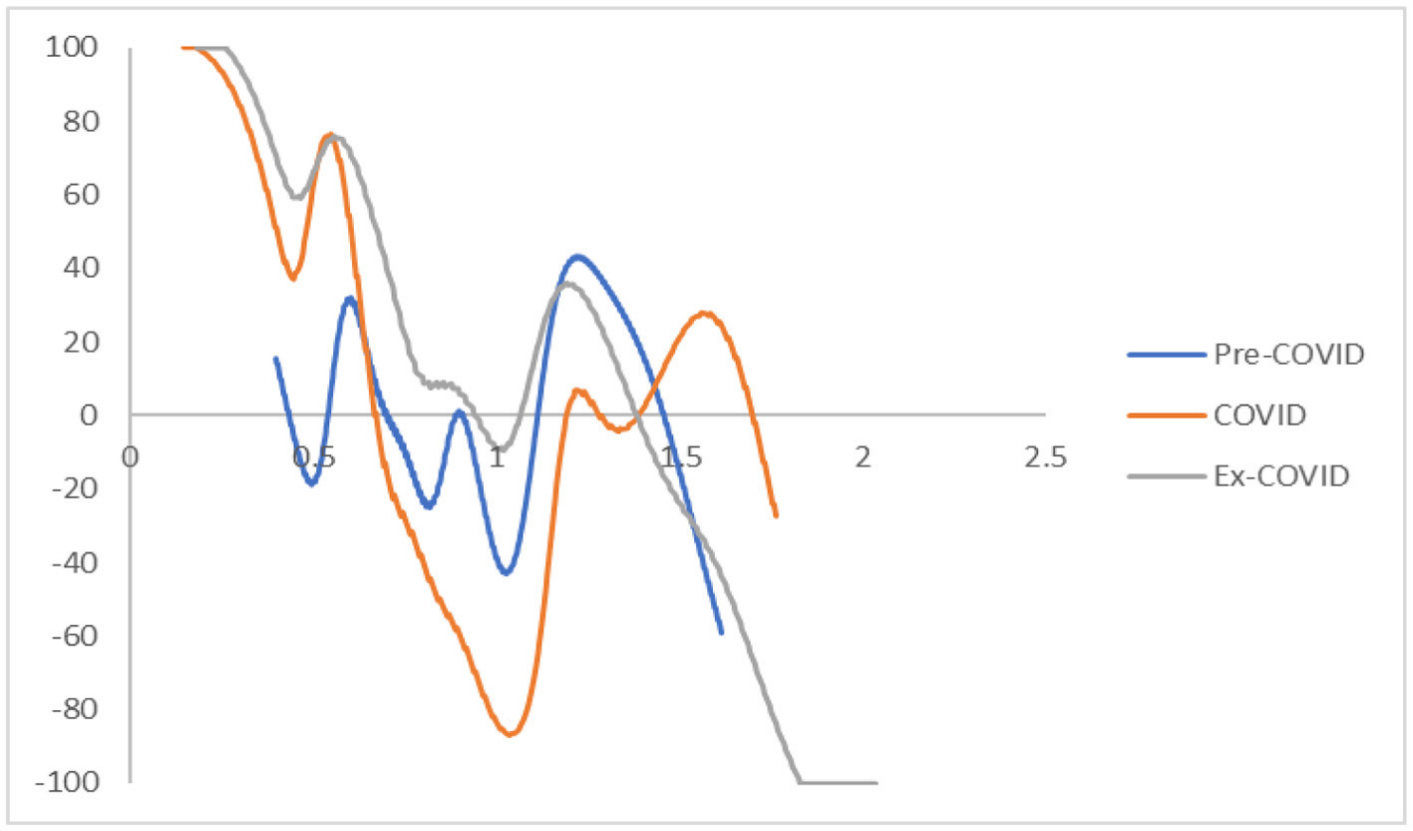
Mobility probability plot (MPP) for the RDIPC of all residents in cities with the shortest duration of Level I ERS with quarterly transitions. Source: authors' calculation. N.B. The vertical axis indicates net upward mobility (%) and the horizontal axis indicates RDIPC values.

The MPP of RDIPC of all residents in cities with a short duration of Level I ERS during the Pre-COVID, COVID, and Post-COVID periods are shown in Figure 8. The MPPs in Figure 8 confirm what can be observed from the transition dynamics. The MPP during the COVID period has negative values when the RDIPC values range from 0.6 to 1.2 and positive values when the RDIPC value is above 1.2 and below 1.7.

**Figure 9:**
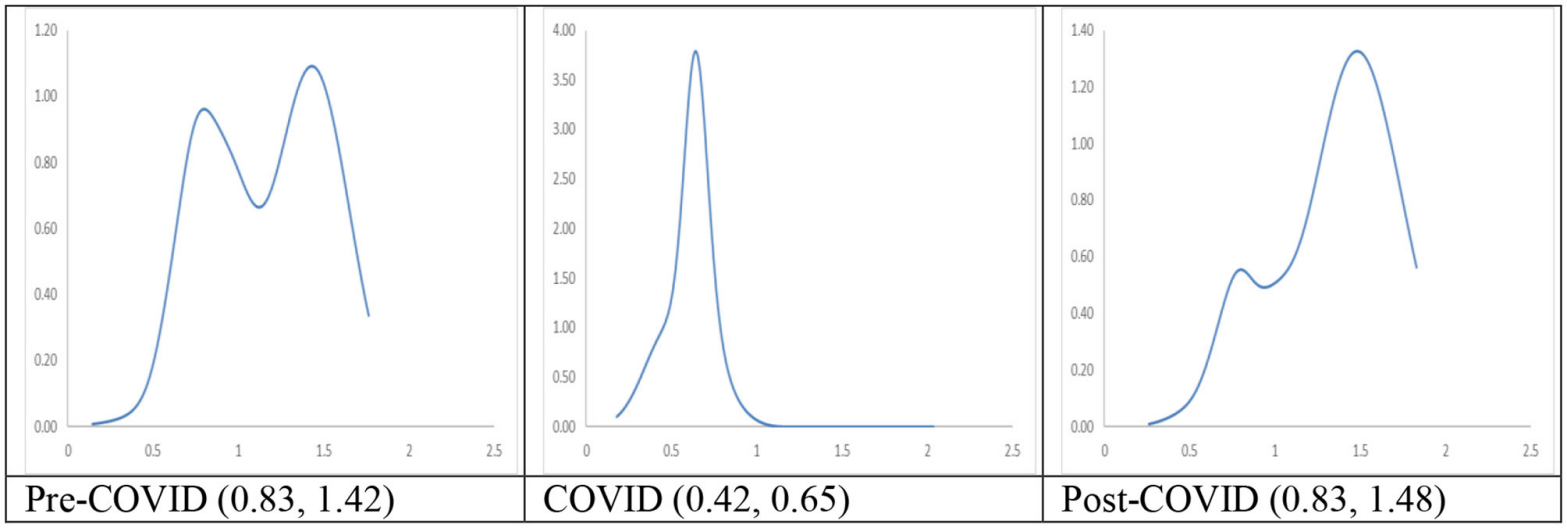
Ergodic distributions for the RDIPC of all cities with short duration of Level I ERS. Source: authors' calculation. N.B. The vertical axis indicates the density of probability, the horizontal axis indicates RDIPC values, and the value of the peaks are in parentheses.

The long-run steady-state ergodic distributions and the MPP of RDIPC of all residents in cities with a short duration of Level I ERS during the Pre-COVID, COVID, and Post-COVID periods are shown in Figure 9. Two peaks at 0.83 and 1.42 can be observed from the Pre-COVID ergodic distribution. The convergence clubs indicate that income disparity exists before the pandemic since a group of cities converges to an RDIPC value less than the country mean, while another group of cities converges to an RDIPC value 1.4 times more than the country mean.

The entire ergodic distribution of the COVID period shifts to the left with a single peak located around the RDIPC value of 0.65. It indicates that most of the cities converge to an RDIPC value which is less than the below-the-average peak in the Pre-COVID ergodic distribution^12^. It can be seen that if the COVID dynamics persist, most cities will converge to RDIPC value which is less than the Pre-COVID level. However, the disparity will disappear during the COVID period due to the upward transition limit, i.e., a city has a positive chance to move upwards. The two peaks of the Post-COVID ergodic distribution are located at RDIPC values of 0.83 and 1.48, respectively. They are almost the same level as in the Pre-COVID case. Thus, it can be concluded from the transition dynamic analysis that the pandemic was under control during the Post-COVID period and the cities with the shortest duration of Level I ERS successfully recovered from the pandemic.

Taken together, our findings show that COVID-19 pandemic yields negative impacts on the average disposable incomes in the 295 Chinese cities. The negative impacts are more substantial in the cities with the longest duration of Level I ERS (the value of the convergence club decreases from 0.93 in the pre-COVID period to 0.78 during the COVID period), probably due to a longer time of economic freeze, and in the cities with shortest duration of Level I ERS (from 0.83 to 0.65). This is because these cities are poorer and more vulnerable to the economic shock. Another consequence of the COVID-19 is that the regional income inequalities would be intensified in some cities if the pandemic persists. The mobility patterns of disposable incomes in the post-COVID period show that most cities in China have recovered from the COVID-19 recession, although the level of disposable income in the cities with longest duration of Level I ERS did not restored to the level in the pre-COVID period (0.83 in the post-COVID period vs. 0.93 in the pre-COVID period). The stringent social distancing policies adopted in China effectively contained the spread of virus in < 1 month in most cities and the economy was restored quickly in the second quarter of 2020. However, a long duration of stringent social distancing policies, e.g., more than 1 month, could generate negative economic impact on cities after the pandemic was under the control (25).

### RDIPC of Urban Residents

As shown in the previous section, the transition dynamics can be fully interpreted from the MPP. Thus, we only discuss the MPPs and the ergodic distributions for the rest of this section.

**Figure 10:**
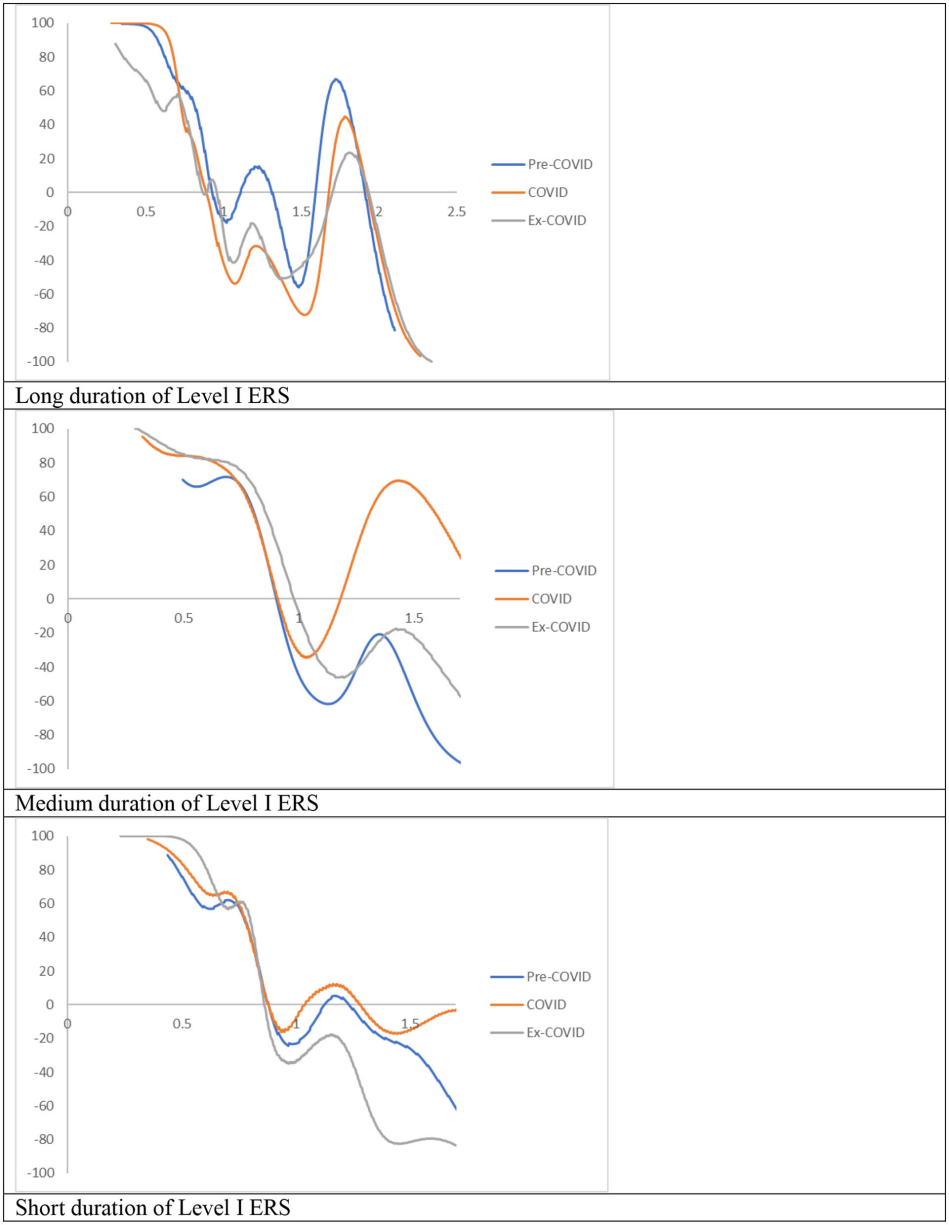
Mobility probability plot (MPP) for the RDIPC of urban residents in cities with long, medium, and short duration of Level I ERS with quarterly transitions. Source: authors' calculation. N.B. The vertical axis indicates net upward mobility (%) and the horizontal axis indicates RDIPC values.

In the MPPs for the RDIPC of urban residents in Figure 10, it can be observed that the Pre-COVID and Post-COVID MPPs share similar shapes. The transition dynamics underlying the MPPs during the COVID periods are distinguishably different from the other two periods. The distinctive features include: First, the COVID MPP for urban residents in cities with the longest duration of Level I ERS is the MPP with the most negative net upward mobility. It indicates that the cities experienced a difficult period to move upward in the distribution during the COVID period. Second, the COVID MPP for urban residents in cities with medium and short duration of Level I ERS share a similar feature, i.e., the tail of the MPP tilted upwards. It indicates that during the COVID period, cities with relatively high RDIPC have a higher chance of moving upward in the distribution. The above features are distinctive for the COVID period and the shape of the MPP reverts to the Pre-COVID case for all durations of Level I ERS. Therefore, it can be concluded that the effects of the pandemics, if any, disappeared or faded away during the Post-COVID period.

**Figure 11:**
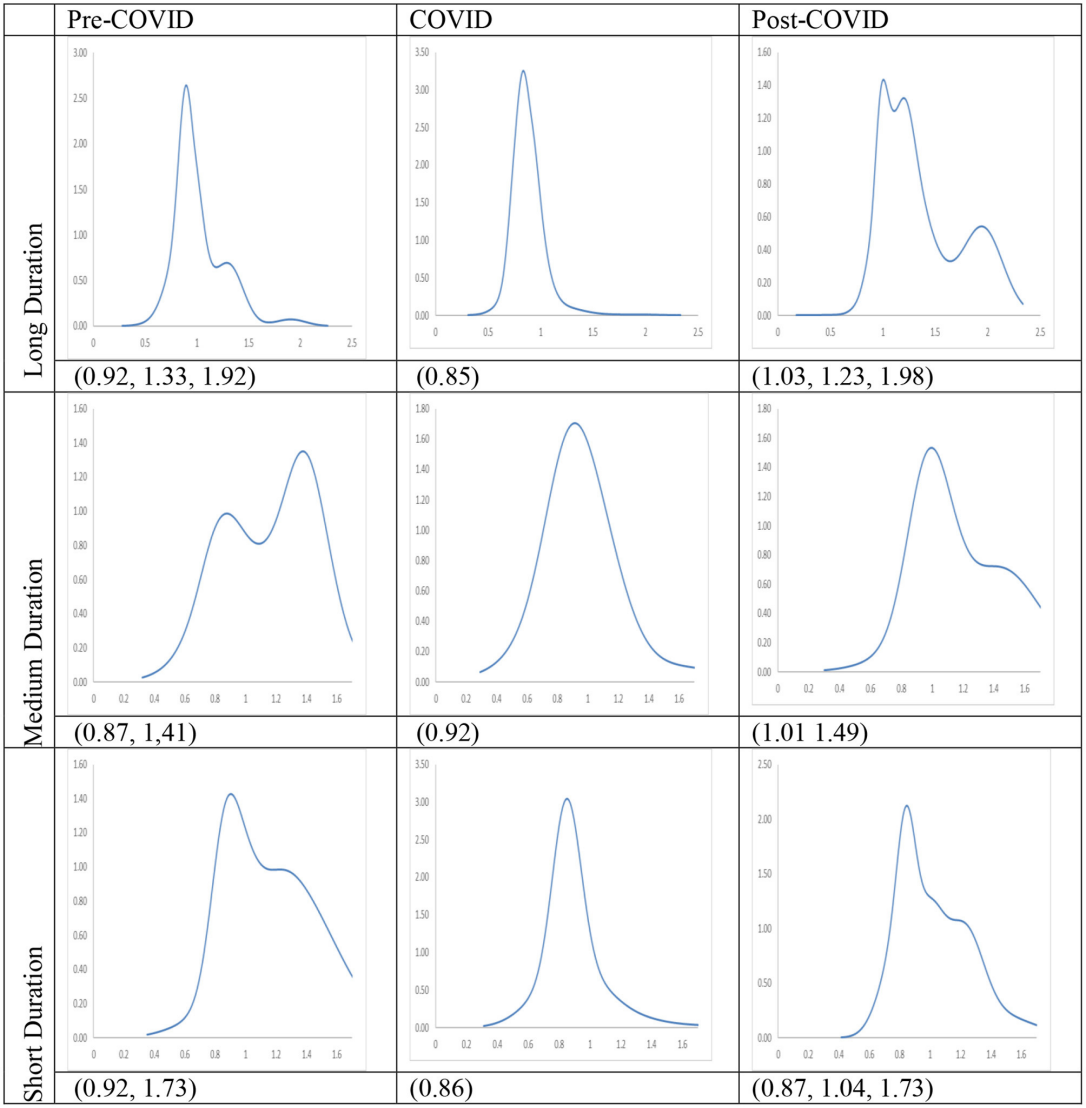
Ergodic distributions for the RDIPC of urban residents in cities with long, medium, and short duration of Level I ERS. Source: authors' calculation. N.B. The vertical axis indicates the density of probability, the horizontal axis indicates RDIPC values, and the value of the peaks are in parentheses.

The transition dynamics underlying the MPPs will eventually translate to the ergodic distributions, as shown in Figure 11. Notably, the Pre-COVID and Post-COVID ergodic distributions have similar shapes whereas the COVID ergodic distribution looks completely different from the other two periods. The peaks in the cities with the longest and shortest duration of Level I ERS are reduced from a higher level in the pre-COVID period (0.92/0.92) to a lower level (0.85/0.86), thus indicating that the COVID-19 and the social distancing policies have negative impacts on the cities in these two groups. Surprisingly, the peak in the cities with medium duration increases slightly. The COVID distributions exhibit unimodal distribution in the three groups while the Pre-COVID and Ex-COVID distributions have multiple peaks. Similar to the aforementioned mentioned observations, all urban residents in cities with long, medium, and short duration of Level I ERS faced an upward transition limit during the COVID period; the regional disparity disappears during the COVID period due to the upward transition limit.

All these ergodic distributions with different durations of Level I ERS shift back to the right and exhibit multiple peaks when the pandemic was over, as shown by the Post-COVID ergodic distributions. The first peaks in the cities with long and medium duration of Level I ERS are 1.03 and 1.01, respectively, which are even higher than the first peaks in the pre-COVID period (0.92 and 0.87). The results indicate that disposable incomes of urban residents in these cities even increase in the second quarter of 2020 due to the economic recovery. In contrast, urban disposable incomes in the cities with short duration of Level I ERS are not restored to the level in the pre-COVID period. Even though these cities did not have as many confirmed cases as cities in the other two groups and implemented stringent social distancing policies in a shorter time, the economy was hit harder than other cities and the recovery was slow. The results also show that regional income inequality appear again in all cities during the post-COVID period, as reflected in the multiple peaks in the ergodic distributions.

### RDIPC of Rural Residents

**Figure 12:**
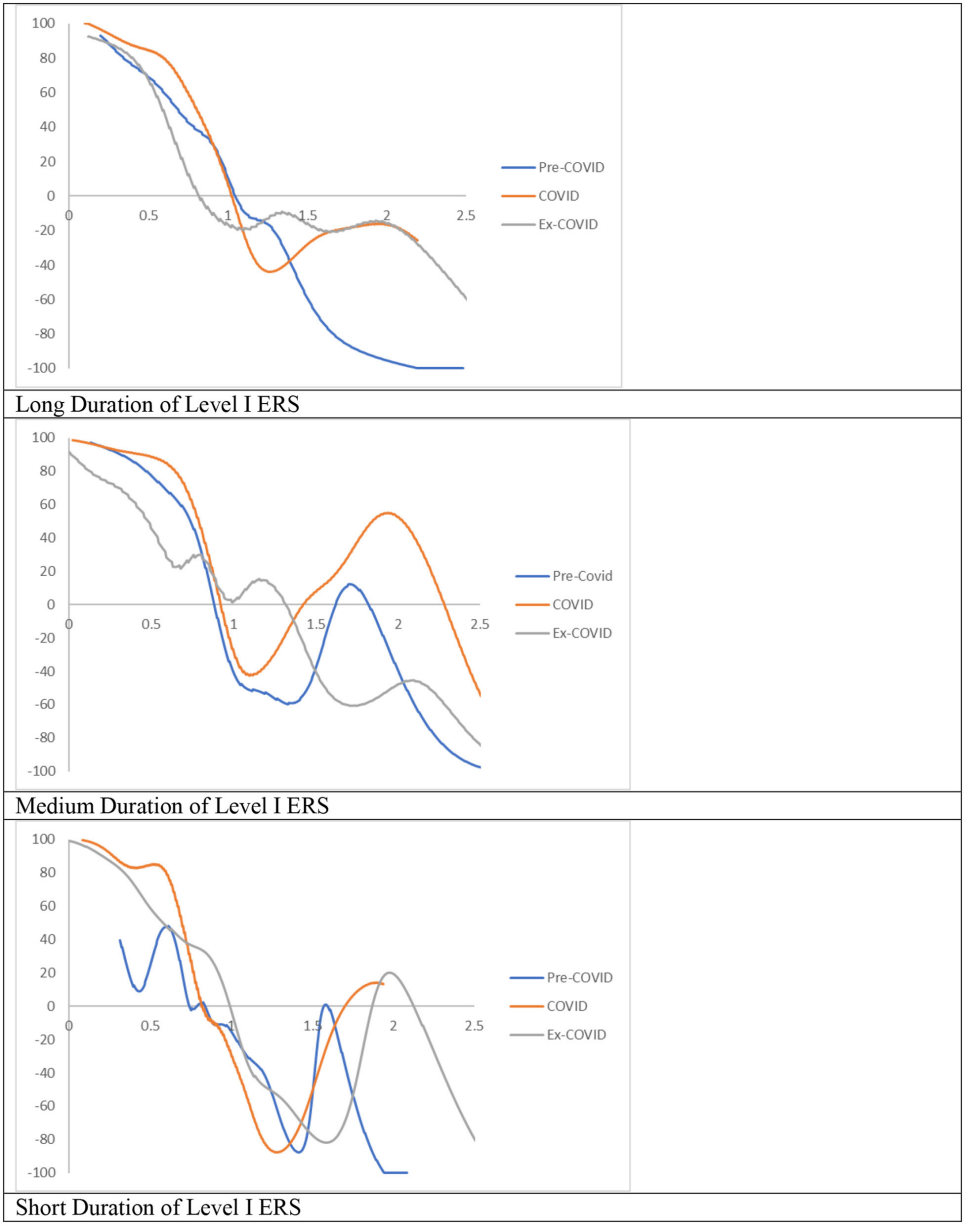
Mobility probability plot (MPP) for the RDIPC of rural residents in cities with long, medium, and short duration of Level I ERS with quarterly transitions. Source: authors' calculation. N.B. The vertical axis indicates net upward mobility (%) and the horizontal axis indicates RDIPC values.

**Figure 13:**
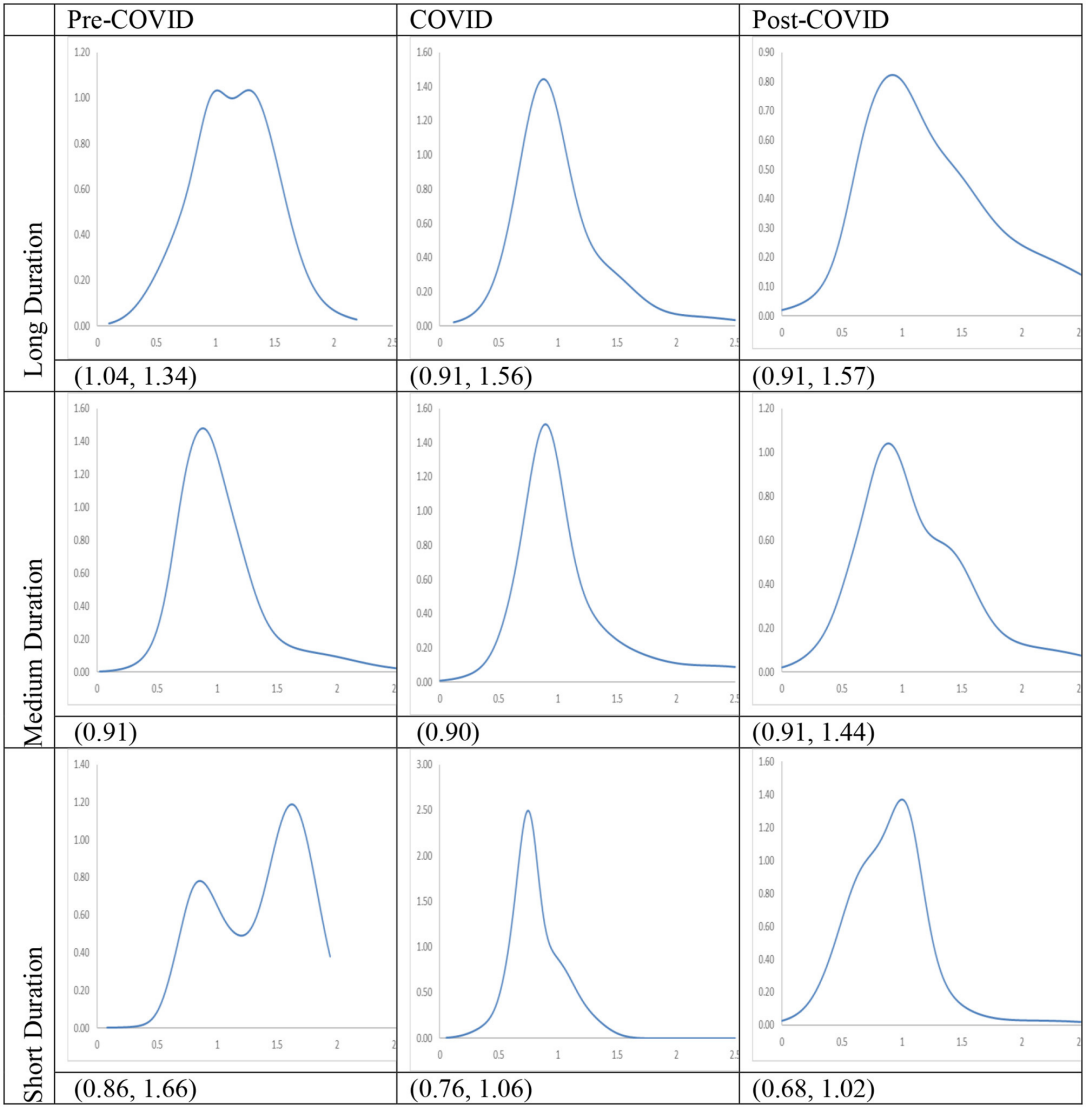
Ergodic distributions for the RDIPC of urban residents in cities with long, medium, and short duration of Level I ERS. Source: authors' calculation. N.B. The vertical axis indicates the density of probability, the horizontal axis indicates RDIPC values, and the value of the peaks are in parentheses.

Recovery from the pandemic is less promising for rural residents, shown in the Figures 12, 13. For cities with a long and medium duration of Level I ERS, two distinctive features can be observed. First, the ergodic distribution shifts to the left due to the pandemic. Second, the tail of the COVID MPP tilted upward. Thus, cities with a relatively high level of RDICPs have a more positive net upward mobility during the pandemic that intensified income disparity during the COVID period. These features remain the same, if not intensified, during the Post-COVID period. The first peaks in the ergodic distributions in the Post-COVID periods are 0.91, which are almost identical to the peaks in the COVID period. Thus, it can be concluded that there is yet to be a full recovery from the pandemic during the Post-COVID period. The regional income inequality, however, is exacerbated as the distances between two peaks become larger (e.g., 0.91, 1.57 in the cities with long duration in the Post-COVID period vs. 1.04, 1.34 in the Pre-COVID period).

For cities with the shortest duration of Level I ERS, it can be observed that the peaks of the COVID ergodic distribution (i.e., 0.76, 1.06) are below the peaks of the Pre-COVID ergodic distribution (i.e., 0.86, 1.66). Additionally, the peaks of the Post-COVID ergodic distribution (i.e., 0.68, 1.02) are also below the peaks of the Pre-COVID ergodic distribution. It indicates that the adverse impacts have been intensified during the Post-COVID period. The COVID-19 pandemic has a lasting adverse impact on the disposable incomes of rural residents in the relatively poor cities, even after the removal of the social distancing policies. One possible reason is that small and medium firms may be shut down during the pandemic and run at partial capacity after the pandemic (32), which cause lower demand for the labor. The rural residents in the western regions are affected more than rural residents in the rich regions as they are more likely to be migrant workers and hence, may be restricted to return due to the social distancing policies in the destination cities in the eastern regions' destination cities.

In sum, if the COVID dynamics persist, the relative disposable income for rural residents in cities with all durations will converge to RDIPC value which is less than or equal to the Pre-COVID level, and the disparity will be intensified due to the pandemic. Unlike urban residents, the impact of the pandemic on rural residents remains unresolved, if not intensified, during the Post-COVID period. Among the three groups of cities, the residents in the cities with the shortest duration of Level I ERS suffered the largest loss from the COVID-19 pandemic, in both urban and rural areas.

## Conclusion

In this study, we examine the impacts of the COVID-19 and the duration of Level 1 ERS on income inequality across 295 cities in China. The distribution dynamics approach is used to analyse the trend and movement of disposable income per capita in a city before the COVID-19, during the COVID-19 pandemic and in the Post-COVID period when the virus was largely contained. The results show that the COVID-19 has significant negative economic consequences: if the COVID pandemic persists, most cities will converge to a level of disposable income which is less than the Pre-COVID level. The regional income disparity will be intensified due to the pandemic in the cities with long and medium durations of Level I ERS. On the other hand, the disparity that appeared in the Pre-COVID period will disappear during the COVID period due to the upward transition limit, i.e., a city has a positive chance to move upwards but has an upward limit. These findings confirm that social distancing policies have significant economic consequences and could exacerbate the regional income inequality (24).

This study also reveals that cities in all the three groups of Level I ERS durations have recovered from the pandemic during the Post-COVID periods. However, recovery from the pandemic is less promising for rural residents than for urban residents, and for cities in the western regions. The economic impact triggered by the pandemic faded away in the urban residents in eastern and central regions; despite this, the impact of the pandemic on rural residents remains unresolved, if not intensified, during the Post-COVID period. In addition, regional income disparity in the rural residents also worsens, especially in the regions with longer Level I ERS duration. The results are consistent with the previous findings that low-income individuals are more severely affected by the economic consequences of the social distancing policies (2, 8).

This study yields several important policy implications. Our findings suggest that stringent nation-wide social distancing policies adopted in China are effective as the economy recovered quickly when the transmission of coronavirus was contained. If a country can flatten the curve in a short time, e.g., several weeks, the economic loss can be limited. The long duration of strict social distancing policy however may intensify the regional income equality. Special policy attention is required for rural regions as the effect of the pandemic could be long-lasting. China has adopted poverty alleviation programme since 2013 to eliminate the extreme rural poverty in 2020, and lifted 100 million rural residents out of poverty. As rural regions, especially those in the western areas, were hit more severely by the COVID-19 pandemic, more efforts should be spent to prevent rural residents slipping back into poverty.

## Data Availability Statement

Publicly available datasets were analyzed in this study. This data can be found at: https://www.ceicdata.com/en.

## Author Contributions

All authors contributed to the conception and design of the study. JS compiled the data and conducted the literature review. WS wrote the discussions. TC prepared the data and conducted the analysis. LW wrote the data preparation process and methodology section. All authors contributed to manuscript revision, read, and approved the submitted version.

## Conflict of Interest

The authors declare that the research was conducted in the absence of any commercial or financial relationships that could be construed as a potential conflict of interest.

## Figures and Tables

**Appendix 1:**
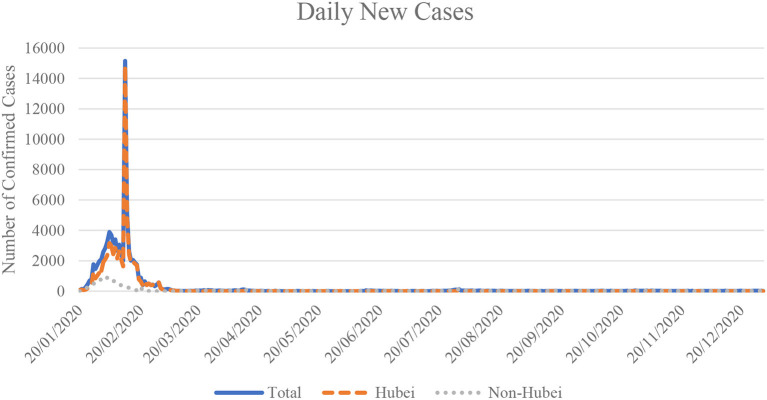
Daily new cases in China. Data source: China CDC.

## Footnotes

^1^According to the statistics at Johns Hopkins University, the numbers of global infected cases and death cases are 121,845,601 and 2,692,235 up to March 19, 2021. See: https://coronavirus.jhu.edu/map.html.

^2^See: https://www.imf.org/en/Data.

^3^We exclude Hong Kong, Macau, and Taiwan in our analysis.

^4^After a massive break of COVID-19 in China from the last week of January 2020, the government adopted aggressive social distancing policies to restrict the spreading of the virus. The cumulative confirmed cases reached a peak on February 5, 2020, and the epidemic curve had been flattened significantly after February 26, 2020. Many cities reported zero new confirmed cases since then.

^5^For instance, a second wave of infections occurred in Beijing with 256 new local cases between June 11, 2020 and June 23, 2020.

^6^Based on the size and severity of causality, China established a four tiers of emergency response situation. The Level I is the top ERS. Tibet was the last province that announced Level I ERS on January 27, 2020.

^7^There are 337 prefecture (and above) cities in 31 provinces in China (excluding Hong Kong, Macau, and Taiwan). Some cities are not included in the sample as the data are not available.

^8^CEIC complies the data of disposable income per capita from Municipal Bureau of Statistics in each city.

^9^Per capita disposable incomes are normally highest in the first quarter of each year as residents could receive extra salary and bonus before Chinese New Year. The statistics also show a significant gap of disposable incomes between urban residents and rural residents.

^10^The figures are consistent with official statistics that the average disposable income in China fell by 3.9% in the first quarter of 2020 but rose by 2.1% in the whole year of 2020. See: http://news.cctv.com/2020/04/17/ARTIpIS9jocjvL5g0dR1haG5200417.shtml; http://news.voc.com.cn/article/202101/202101181012395850.html.

^11^Our analysis focuses on the quarters before the COVID-19 pandemic, during the pandemic, and after the pandemic in China, as the virus was effectively contained in the first quarter of 2020. Table 3 shows that disposable incomes after the pandemic quickly bounce back to a higher level than the same quarters in the previous year since 2020Q2.

^12^Note that the MPP during the COVID period has positive values when the RDIPC value is above 1.2 and below 1.7 and has an INTERSECT at an RDIPC of 1.7. However, the positive upward mobility fails to translate into a convergence club in the long run, since cities with RDIPC > 1 can hardly be observed from the COVID ergodic distribution. This failure can be explained in the following way: since the MPP shows only the probability of transition without showing the transition value, it is possible that some cities with positive upward mobility at RDIPC of 1.7 transit to a slightly higher position. Other cities with RDIPC of 1.7, on the other hand, transit to an exceptionally low position in the distribution. If this is the case, cities will not be able to congregate at the INTERSECT with RDIPC of 1.7. Thus, it can be concluded that during the COVID period, cities with RDIPC higher than the country mean experienced an upward transition limit, i.e., the city has a positive chance to move upwards. However, the upward movement has an upward limit; it cannot transit to a high level of RDIPC.
